# Knowledge and perceptions about diet and physical activity among Sri Lankan adults with diabetes mellitus: a qualitative study

**DOI:** 10.1186/s12889-015-2518-3

**Published:** 2015-11-23

**Authors:** P. Ranasinghe, A. S. A. D. Pigera, M. H. Ishara, L. M. D. T. Jayasekara, R. Jayawardena, P. Katulanda

**Affiliations:** Department of Pharmacology, Faculty of Medicine, University of Colombo, Colombo, Sri Lanka; Medical Education Development and Research Centre, Faculty of Medicine, University of Colombo, Colombo, Sri Lanka; Institute of Health and Biomedical Innovation, Queensland University of Technology, Brisbane, QLD Australia; Diabetes Research Unit, Department of Clinical Medicine, Faculty of Medicine, University of Colombo, Colombo, Sri Lanka

**Keywords:** Diabetes, Diet, Physical activity, Perception, Qualitative study, Sri Lanka

## Abstract

**Background:**

Diabetes mellitus (DM) is a rapidly growing health concern in Sri Lanka. Diet and physical activity are important modifiable risk factors affecting the incidence, severity and management of DM. The present study aims to evaluate the knowledge and perceptions about dietary patterns and physical activity among a group of adults with DM in Sri Lanka using qualitative research methods.

**Methods:**

Fifty adults from a cohort of diabetic patients attending the medical clinics at the National Hospital of Sri Lanka were invited for the study. Data were collected via 10 Focus Group Discussions. Verbatim recording and documenting emotional responses were conducted by two independent observers. Directed content analysis of qualitative data was done with the help of NVIVO v10.0.

**Results:**

Mean age was 61.2 ± 9.9 years and 46 % were males. Mean duration of diabetes was 10.4 ± 7.5 years. All were aware of the importance of diet in the management of DM. But most had difficulty in incorporating this knowledge into their lives mostly due to social circumstances. The majority described a list of ‘good foods’ and ‘bad foods’ for DM. They believed that ‘good’ foods can be consumed at all times, irrespective of quantity and ‘bad’ foods should be completely avoided. Many believed that fruits were bad for diabetes, while vegetables were considered as a healthy food choice. The majority thought that there were ‘special’ foods that help to control blood glucose, the most common being curry leaves and bitter-gourd. Most study participants were aware of the importance of being physical active. However, there was lack of consensus and clarity with regards to type, duration, timing and frequency of physical activity.

**Conclusions:**

Despite understanding the importance of dietary control and physical activity in the management of diabetes, adherence to practices were poor, mainly due to lack of clarity of information provided. There were many myths with regards to diet, some of which have originated from health care professionals. More evidence is needed to support or refute the claims about ‘special’ foods that the participants believe as being good for diabetes.

**Electronic supplementary material:**

The online version of this article (doi:10.1186/s12889-015-2518-3) contains supplementary material, which is available to authorized users.

## Background

Diabetes mellitus is a leading cause of morbidity and mortality worldwide, with an estimated 80 % of the affected population living in developing countries like Sri Lanka [1]. Sri Lanka is a middle income developing country in the South Asian region with a population of nearly 21 million [2]. Prevalence of diabetes in Sri Lanka, which was around 2.0 % in the early nineties has increased by about five-fold during the last two decades [3]. It is estimated that over 2 million people are suffering from diabetes and nearly 4 million adults have dysglycaemia [3]. Diabetes results in a host of macro- and micro-vascular complications. Hence, the direct and indirect health care expenditure and economic impact due to diabetes and its complications is likely to be exponential in a developing country like Sri Lanka. It is well known that South Asians are at an increased risk of developing diabetes, with higher risk of complications, morbidity and mortality [4, 5]. Changes in dietary patterns and physical activity levels in the region coupled with the genetic predisposition is probably one of the main reasons driving the current diabetes epidemic in the South Asian region [6].

Sri Lanka has a higher proportional prevalence of type-2 diabetes (>90 %), while the prevalence of autoimmune type-1 diabetes is rare [7]. The causes of type 2 diabetes are multi-factorial. Diet and physical activity are important modifiable risk factors, that play a central role in the incidence, severity and management of diabetes [8]. Energy intake in excess of bodily requirement resulting in obesity is a major driving force behind escalating type-2 diabetes epidemics worldwide [9]. Studies have shown that quantity and quality of the diet are both independently associated with the risk of diabetes [9]. Nutrition therapy is recommended for all people with type-2 diabetes as an essential component of the overall management plan [10]. There are numerous guidelines on nutritional recommendations for patients with diabetes mellitus, published by many leading organizations around the world, including the American Diabetes Association (ADA) [10]. In its position statement, the American Dietetic Association strongly emphasizes that the total diet or overall pattern of food eaten is the most important focus of a healthful eating style. All foods can fit within this pattern, if consumed in moderation with appropriate portion size and combined with regular physical activity [11]. Studies have shown that in most situations, nutrition messages are more effective when focused on positive ways to make healthful food choices over time, rather than individual foods to be avoided [12]. Many developing nations, including Sri Lanka are currently experiencing rapid economic and social development which promotes over-nutrition and positive energy balance [9]. Even in Sri Lanka, traditional dietary patterns are changing, with the population adapting to more industrialized and urban food environments. Sri Lankans are known to consume a diet with high starch content, with comparatively lesser amounts of fruits and vegetables [13]. Most Sri Lankans, including patients with diabetes, obtain over 70 % of their energy from carbohydrates [14].

Physical activity is important in the management of diabetes. The ADA recommends two types of physical activity for patients with diabetes, aerobic exercises and strength training. The ADA recommends 30 min of moderate-to-vigorous intensity aerobic exercise at least 5 days a week or a total of 150 min per week, and some type of strength training at least two times per week in addition to aerobic activity [15]. Increased physical activity reduces the risk of diabetes, whereas sedentary behaviour is known to increase the risk [16]. Even among Sri Lankan adults physical inactivity is known to be associated with obesity, diabetes, hypertension and metabolic syndrome [16, 17]. Increased mechanization has displaced physical activity over the last century in industrialized nations, a trend that is increasingly observed in developing countries as well [9]. Evaluating patients’ perceptions and knowledge about healthy lifestyles (diet and physical activity) would help in the development of evidence based interventions, aimed at curbing the ongoing epidemic of diabetes. Qualitative research methods are an invaluable tool in evaluating patient perceptions. They help to generate a wide array of different perceptions/comments/observations from a vast population over a short period of time, in contrast to a structured questionnaire based study.

Although a small country, Sri Lanka has a high disease burden related to diabetes. Furthermore, there is a larger population of Sri Lankans living outside the island as immigrants and workers in countries like Canada, UK and other European countries, Australia and USA (nearly 2 million), who also carry the same disease risks. Hence diabetes and its associated factors are not confined to one country, but is rapidly becoming a global problem. In addition since Sri Lanka is an island, although a part of South Asia it has unique socio-cultural issues not seen in other South Asian countries. For instance, Sri Lankans consume mainly rice, but in other regional countries wheat-based meals are more common [13]. Protein consumption pattern is also different in Sri Lanka. Fish intake is high due to high availability and due to Buddhist religious dominance. In other countries in South Asia it is different, for example beef consumption is rare in India due to Hindu influences and pork consumption is very rare in Pakistan and Bangladesh due to Islamic influences. Currently there are no studies evaluating patients’ perceptions about diet and physical activity among Sri Lankan adults with diabetes. Therefore, our study adds unique data on dietary perceptions and practices of a main group of South Asians which has not been discussed in the literature before. The present study aims to evaluate the knowledge and perceptions about dietary patterns and physical activity, among adults with Diabetes Mellitus from Sri Lanka using qualitative research methods.

## Methods

### Study population and sampling

Fifty adults were recruited from a cohort of patients with diabetes mellitus attending the Medical Clinics of the University Medical Unit at the National Hospital of Sri Lanka (NHSL) during a period of 2 months from September to October 2013. Situated in Colombo, the NHSL is the largest tertiary care hospital in Sri Lanka. Sixty adults (≥18 years), with a confirmed diagnosis of diabetes for more than 6 months were invited for the study. Patients with other co-morbid diseases where diet and/or physical activity plays a role as a risk factor or is an integral part of treatment were excluded (e.g. ischemic heart disease, hypertension, chronic kidney disease and chronic liver disease).

The medical clinics of the University Medical Unit are held twice per week, each day there are about 200 patients with medical illnesses visiting the clinic for follow up. These patients are given a clinic number on the first come basis and they come to clinics usually once per month. In order to choose the sample for our study, we used a random sampling technique. In each week we selected a random day to visit the clinic. On each day of study we first selected all patients satisfying the above inclusion criteria from the daily clinic register. These patients were then arranged in ascending order according to their clinic number, and five patients were selected on each day by using simple random sampling. Informed written consent was obtained from each study participant, after giving them adequate time (1/2 to 1 h) to ask questions and clarify doubts about the research. Ethical approval for the study was obtained from the Ethics Review Committee, Faculty of Medicine, University of Colombo, Sri Lanka (EC-13-038) and institutional approval was taken from the National Hospital of Sri Lanka.

### Data collection

Data were collected using Focus Group Discussions (FGDs). On each day a single FGD was be conducted by a facilitator (RJ), among five patients (P_1_-P_5_) with diabetes. Altogether there were 10 FGDs conducted (FGD_1_-FGD_10_). The FGD was conducted using a set of open-ended semi-structured questions to guide the participants and to keep uniformity between the different focus groups (Additional file 1). The questions were mainly focused on the diet and physical activity. The duration of each FGD was 1 h, and each FGD was tape recorded after obtaining consent from the participants. The FGD was conducted in Sinhalese, since all participants were fluent in Sinhalese. Two independent observers (ASADP and MHI) at each FGD were responsible for verbatim recording/transcribing and documenting emotional responses. One observer transcribed the verbal responses and the other observer noted down the emotional responses of the group/individuals. The verbal responses were transcribed in participants’ own words, while emotional responses of individuals and the entire group to different questions were also recorded. At the beginning of each FGD, the following socio-demographic details were recorded from each participant; age, gender, area of residence, marital status, occupation, level of education and duration of diabetes. The facilitator/moderator was a female health care practitioner and a trained researcher, with experience in conducting focus group discussions. The facilitator provided guidance, maintained focus, stimulated constructive debate, regulated the flow of discussion and ensured time adherence, while maintaining a neutral stance on contents of discussion. Data collection was stopped when data saturation was reached and no new themes emerged.

### Data analysis

Directed content analysis of qualitative data was conducted with the assistance of NVIVO v10.0 (QSR International, Southport, UK). The topics/themes were selected prior to the analysis and participants’ responses were grouped under these topics/themes. The topics selected on diet were; dietary perceptions, frequency of meals, quantity of diet, sugars and sweeteners, starch and carbohydrates, proteins, fruits and vegetables. In physical activity; duration and timing of physical activity, types of physical activity, and barriers to engage in physical activity were the topics selected.

The facilitator and two observers were entrusted with the task of providing an analysis of verbal and non-verbal responses of participants in each of the FGDs within 24 h of conclusion of each FGD. The facilitator and two observers of each FGD analyzed their respective FGD by using their notes and the tape recorded data. This was done collectively immediately after the conclusion of the FGD. They were expected to note down all facts mentioned by the participants by corroborating their notes with the tape recording. Each FGD produced one such complete document, and these documents were collectively analysed at the end of all FGDs by the research team. The data were initially transcribed in Sinhalese, which was the native language of study participants. Subsequently the cumulative FGD data were translated to English by two independent translators. The independent translations were compared by the research team with the help of the translators and the final document was prepared after an iterative consensus process.

## Results

### Sample characteristics

Sample size was 50 (response rate – 83.3 %), with a mean age of 61.2 ± 9.9 years (range 38–82 years) and 46 % were males (*n* = 23). All were urban and semi-urban residents from Colombo. The majority of the population were Sinhalese in ethnicity (72.0 %, *n* = 32), while 20.0 % were Muslims (*n* = 10). Mean duration of diabetes among the study participants was 10.4 ± 7.5 years (range 1–35 years). The majority were educated up to grade 6–11 in school (*n* = 19, 38.0 %), unemployed (*n* = 32, 64.0 %) and had a household income < LKR 20,000 (US$ <154) per month (*n* = 28, 56.0 %).

#### Dietary perceptions

Almost all (n-45) study participants were aware that diet plays an important role in the management of diabetes mellitus. Resembling this general idea, participants stated that;

*“Diabetes is a very dangerous disease leading to foot ulcers, most of the times I think that I should be very cautious about the food and drinks I take.”* (FGD_1_; P_2,_ 45 years, Female)

*“It is very essential to be cautious about the food I eat and drinks I take. It is not good to disregard the role of diet in diabetes management even for a moment. Spoon is in our hand and we have the control.”* (FGD_9_; P_5_, 52 years, Female)

“*Sometime, actually most of the times, according to the circumstances I tend to eat and drink sweets and go out of the dietary advice given by the doctors, even though I know that it is very important.”* (FGD_10_, P_1_, 50 years, Male)

But most of them (n-35) had difficulty in incorporating the dietary advice given by medical personnel to their lives, despite knowing the impact of the diet and sometimes even when having a good knowledge on what they should practice. Social circumstances had a major impact on the difficulties in incorporating dietary advice into the practical life. One of the participants expressed this as;

*“I know it is bad but I have to eat what my daughter is cooking.”* (FGD_3_; P_5_, 65 years, Male)

However, few participants’ (n-8) social issues had actually led them to adhere more to the correct dietary practices;

“*In my case I am very cautious of the diet I take. If I become sick, all the family members will be affected. Husband will suffer more than me.”* (FGD_3_; P_2_, 41 years, Female)

Some participants (n-12) have started being more cautious about their diet only after developing complications (according to their perception) of diabetes;

“*I suffered a lot due to a urinary tract infection 2–3 months back. Now I strictly adhere to the dietary advice given by doctors, because I believe I can get rid of these complications if I strictly control my diet”* (FGD_7_; P_1_, 51 years, Female)

People were very interested and enthusiastic in searching for more information regarding correct dietary practices for diabetic patients as they perceived dietary control as the key to the blood sugar level control;

“*I am always on the lookout for more information on dietary advice for diabetic patients through books, journals and television programmes.”* (FGD_3_; P_3,_ 57 years, Male)

Few patients (n-7) had their own attitude and perception on the importance of diet and dietary practices in diabetes management. One of them highlighted a self-exploratory approach in identifying the appropriate diet for oneself;

*“I am completely aware that diet plays a major role in the management of diabetes mellitus. But I do not change my dietary practices just depending on the others’ comments. I experiment on myself, how my body responds to different types of foods. I have a glucometer. As an example some people say ‘Del’ (bread fruit) is bad for diabetic patients and once I consumed ‘Del’ (bread fruit) and checked blood sugar with the glucometer and got to know that it is not increasing my blood sugar level. So, my opinion is different patients’ bodies respond differently to various foods which are categorized as good/bad for diabetes. So we should have the ability to identify the response of our own body to those foods.”* (FGD_9_; P_5_, 52 years, Female)

The majority of respondents (n-32, 64 %) came up with a list of “good foods and bad foods” for management of diabetes. Sweet foods, milk products, white colour starch and fruits were widely considered as bad foods. They were with the opinion that ‘good’ foods can be consumed at all times, irrespective of quantity and ‘bad’ foods have to be completely avoided.

### Frequency of meals

Few participants (n-11) mentioned the frequency of meals in relation to diabetes mellitus and very few (n-3) emphasized the importance of taking meals at the correct time.

*“We need to take meals at the correct time, If not I feel dizzy.”* (FGD_7_; P_3_, 62 years, Male)

Some (n-6) had increased the number of meals whilst restricting the individual portion sizes;

*“I was advised to take about 5 meals, but not to have a full stomach.”* (FGD_4_; P_2_, 75 years, Male)

### Quantity of diet

Few people (n-13) perceived that reducing portion sizes was the most important step to achieve glycaemic control, leading to reduction in their overall food intake;

*“After the diagnosis of diabetes, I am eating only half of the amount I usually eat, as it is very important to reduce the size of each meal.”* (FGD_3_; P_2_, 41 years, Female)

However, restriction of the portion sizes has led to problems in some of the patients (n-6);

*“I am eating less but, I am getting hungry always and I find it very difficult to reduce the amount I eat.”* (FGD_7_; P_3_, 57 years, Male)

### Sugar and sweeteners

Most of the study participants (n-36, 72 %) believed that diabetes is caused by their high ‘sugar’ intake earlier in life and hence they completely avoided eating sugar. Nearly all participants (n-47) had been advised by health care personnel to avoid sweets at all times. One participant expressed her view on sugar as, “sugar is toxic”. However sugar alternatives (e.g. dates) were consumed at normal or even higher quantities by them, without restrictions.

Some of them (n-18) had great difficulty in controlling their desire to eat sweets, despite knowing the adverse health outcomes.

*“Cannot eat sweets, very fond to eat… But afraid… I don’t know why I do feel such a craving for sweets… I think all diabetic patients feel the same… It is an uncontrollable craving for toffees, ice cream…”* (FGD_8_; P_1_, 49 years, Female)

Some people (n-13) had problems with cutting down on sugar consumption due to their life style.

*“I am a labourer and I frequently drink tea with sugar from small boutique shops around. I feel it as a nuisance to ask for a tea without sugar every time.”* (FGD_2_; P_2_, 46 years, Male)

Some (n-8) were afraid of the discrimination and did not want others to know that they have diabetes.

*“I am working in an office. I do not like others to know I’m having diabetes. Hence if any sweets are offered I have to eat.”* (FGD_3_; P_4_, 53 years, Female)

Some participants (n-12), especially working people had cut down on the number of cups of tea consumed per day. Some (n-7) had chosen products that they believed to have low sugar content;

*“I don’t eat biscuits other than cream cracker. I think they are good for the diabetic patients as they are not sweet in taste.”* (FGD_6_; P_1,_ 66 years, Female)

### Starch and carbohydrates

Rice is the staple food consumed in Sri Lanka. Study participants were cautious about the type of rice they consume. They had the general impression that, red rice and long grain rice varieties are particularly good for diabetic patients;

*“Before the diagnosis, we consumed only white rice and after the diagnosis, we started having red rice at least few days per week.”* (FGD_10_; P_1_, 50 years, Male)

*“At the initial stage of diagnosis I had only red rice for all 3 meals. Then I changed the type of rice to big seed rice. Any way I do not like eating ‘Samba’ rice” (polished short-grain rice).* (FGD_3_; P_4_, 53 years, Female)

*“After the diagnosis we did not buy ‘samba rice’. The whole family started consuming red rice. I think it is good for my children too.”* (FGD_2_; P_3_, 57 years, Male)

Social impact was an important determinant of the staple meal they consume;

*“Daughter cooks ‘Keeri Samba (polished short-grain rice)’ for every meal. They are not changing it because of me. I have to eat what they give me.”* (FGD_3_; P_5,_ 65 years, Female)

Bread and other wheat flour products also hold a major place in the Sri Lankan diet as many people are used to consuming these products for either dinner, breakfast or both. They believe that eating wheat flour has an association with the development of diabetes. Participants’ ideas on wheat flour products were as follows;

*“I myself suspected that I am having diabetes mellitus and went to a doctor and got to know that I do have diabetes mellitus. I was retired when I was diagnosed. Actually my retirement was a great help for me for the diet control. When I was working, I used to eat bread for both breakfast and dinner. But now I have totally omitted bread in my meal list.”* (FGD_1_; P_5_, 71 years, Female)

### Fruits and vegetables

Many of the participants (n-35) believed that fruits were not good for diabetic patients. Some of their views on fruit consumption were;

*“I have completely avoided all fruits, except for those containing fibre because certain amount of sugar gets absorbed to body from fruits.”* (FGD_7_; P_1,_ 57 years, Female)

Fruits were generally considered as an unhealthy food group by diabetes patients due to their sweet taste. Some (n-11) had received dietary advice from medical practitioners to avoid fruits such as banana and papaya. Some patients (n-7) were asked to eat unripe or half ripened fruits. However, some (n-14) were not aware that fruits have a great influence on the blood glucose level and they were freely consuming fruits without any restrictions;

*“Still I am having a lot of fruits especially in the seasons like ‘Rambutan’ (Nephelium lappaceum) and ‘Mangusteen’ (Garcinia mangostana). I am used to having about 8–10 rambutan and 3–4 mangusteen per day during the season as we have those trees in our home garden.”* (FGD_8_; P_3_, 48 years, Male)

Some people (n-16) who were used to having a lot of fruits have cut down on their fruit consumption due to medical advice;

“*I also used to have many fruits per day, as an example 8–10 well riped bananas per day. After the diagnosis I reduced the amount to 3 half ripened bananas per day.”* (FGD_7_; P_1_, 51 years, Female)

Most of them (n-31) had the idea that fruits should be consumed in the half-ripened state and most had acquired this knowledge from medical personnel:

*“Doctor said to eat half ripen papaw, and not to eat ripen ones.”* (FGD_6_; P_2_, 66 years, Female)

Although vegetables were considered as a healthy food choice, knowledge on the amount of intake and variety was poor. Some (n-9) believed carrot and beetroots are contraindicated for diabetes. Some (n-7) had cut down or omitted on specific types of vegetables like beetroot, carrot, ash plantain and potatoes. Most of their restrictions were due to the advice from different health personnel at different times. Some (n-15) had taken food restriction decisions depending on what other people say, messages conveyed through papers and journals. Some believed that boiled vegetables are good for diabetes.

*“I think Cucumber, brinjal salad and boiled vegetables are good for diabetes.”* (FGD_8_; P_2_, 55 years, Male)

Some (n-11) had the belief that if they take raw vegetables without boiling or tempering, it is better for the diabetic patients. ‘Karavila’ (Bitter Gourd) was also considered to be good by many (n-22) and most of them were consuming it either as kanji (porridge) or a cooked vegetable. Dark green leafy vegetables were widely considered as a healthy food;

“*I purposefully increased the amount of green leaves in my meal. Increased the amount of vegetable intake like ‘Kankukn’ (Ipomoea aquatic) taken from home garden.”* (FGD_2_; P_3_, 57 years, Male)

### Protein

Most of the participants (n-37) did not express specific views or did not have a clear idea on protein intake. The few (n-13) who talked about this topic had various ideas. Some (n-7) thought they should stop meat consumption; “Doctors asked me not to eat meat, so I don’t eat.” Some (n-5) had the idea that “small fish is better than large fish.”

### Other

Most participants (n-27) were with the opinion that there were special food items that help to control blood glucose in addition to medication. Fenugreek Seeds (‘Uluhal’), *Coscinium fenestratum* or yellow vine (‘Venivel gata’), Curry leaves and powder, bitter-gourd, passion fruit leaves, finger millet, jack fruit leaves, wood apples, *Scoparia dulcis* (‘Wal kottamalli’) porridge, dried night jasmine flower, *Wattakaka volubilis* leaves (‘Anguna kola’), *Costus specious* (‘Thebu’) leaves and Ceylon olive fruit (‘Veralu’) were some of the special foods which participants believed had a significant effect on reducing blood sugar;

*“Bitter-gourd juice reduces blood sugar very quickly.”* (FGD_2_, P_2_, 46 years, Male)

*“I have increased bitter gourd consumption as many people are saying it is good for diabetes. I am taking it once- twice per week.”* (FGD_6_, P_1_, 66 years, Female)

*“I take yellow wine (‘venivel geta’) regularly as it is told to reduce blood sugar.”* (FGD_7_, P_5_, 52 years, Male)

Among these foods, curry leaves and bitter-gourd were the most common foods considered by participants to reduce blood glucose. Curry leaves were commonly consumed as porridge and bitter gourd was commonly consumed as a salad.

#### Physical activity

Most people (n-41) were aware that physical activity was important for glycaemic control;

“*According to my knowledge physical activities makes our bodies healthy. Also it reduces sugar level in the blood.”* (FGD_1_; P_1_, 47 years, Female)

*“I have heard when we are sweating from physical activity, it decreases our blood sugar.”* (FGD_2_; P_1_, 52 years, Female)

*“As we grow old, our organs become more and more inactive. Hence physical activity is essential to keep them at an optimal level.”* (FGD_3_; P_2_, 41 years, Female)

### Duration and timing of physical activity

Many (n-37) had received advice on physical activities from various health care personnel either through direct conversations or via various media. But very few stated the advice they received specific to the duration of physical activity:

“*I was told to walk for 5 min after the meals by my nutritionist.”* (FGD_4_; P_2_, 75 years, Male)

Some of the participants (n-17) engaged in walking as a physical activity, stating that they walked for about one-half to 1 h;

*“I walk speedily for about 30 min till my body feels it…”* (FGD_2_; P_2_, 46 years, Male)

*“I am walking for 1 h daily.”* (FGD_2_; P_1_, 52 years, Female)

*“I walk 30 min per day and at least 4 days per week like that.”* (FGD_3_; P_4_, 53 years, Female)

However, most of the participants (n-29) did not have a clear awareness on duration of physical activity. Among the very few who engaged in physical activity regularly, most mentioned afternoons as the preferred time;

*“I am going for a walk in mornings and afternoons.”* (FGD_1_; P_4_, 41 years, Male)

In contrast few participants (n-5) stated that they do not have a specifically allocated time for physical activity but engage in it as and when time permits;

*“I do engage in physical activity as time allows. If I’ll try to follow a time table, I may never be able to do any physical activity.”* (FGD_3_; P_2_, 41 years, Female)

### Type of physical activity

Walking was the most common type of physical activity amongst the study participants. However, different participants had different views with regards to the amount of physical activity they should be engaged in;

*“I walk about ¼ miles every day.”* (FGD_5_; P_1_, 46 years, Female)

“*I walk about 2–3 km daily.”* (FGD_7_; P_2,_ 39 years, Male)

*“I walk 2 km per day.”* (FGD_1_; P_3_, 47 years, Male)

Cycling was another type of physical activity mainly engaged in by male participants. Some (n-7) of the patients had increased the physical activity level in relation to the normal daily activities after the diagnosis of diabetes mellitus;

“*I am a house wife. So since before the diagnosis I always did all house hold work. Washing, cooking, shopping… etc. But after the diagnosis I deliberately increased doing the physical activities which makes me sweat. I never rest at home. Always I do something. I work in the garden and in our paddy field. Also I have to walk for about 30–40 min per day for 2–3 days per week when I have to take my kids to classes. But I am not walking as a physical activity purposefully.”* (FGD_2_; P_5_, 45 years, Female)

*“I sweep the garden for 2–3 h every morning till I am drenched in sweat. I always walk as time allows and I make the opportunities related to my daily activities like walking to the nearby boutique.”* (FGD_1_; P_2_, 45 years, Female)

But a considerable number of people (n-18) were satisfied only with the house hold work they are regularly engaged in. They believed that those activities are adequate and that there is no need for additional physical activity*;*

*“I am doing only the household work. I think those are enough as physical activity.”* (FGD_6_; P_3_, 53 years, Female)

### Barriers to engage in physical activity

Several participants (n-19) mentioned some barriers preventing them from engaging in regular physical activities. Some of them (n-9) felt that diabetes and its co-morbidities prevented them from engaging in physical activity;

*“Physical activities are the biggest problem I have. After the diagnosis of diabetes mellitus I feel like I have become very weak. I am usually a hard working person. I work in my garden daily. But I am not sure what will happen when I do the same activity level while taking reduced portions size. I am becoming thin gradually. I do not know how to maintain my build.”* (FGD_4_; P_2_, 75 years, Male)

*“Very difficult to do physical activity as I develop high pressure and wheezing with physical exertion.”* (FGD_7_; P_1_, 51 years, Female)

“*I got a chest pain few years back. Then doctors told not to exert too much. I’m afraid physical activity would bring a heart attack and therefore I am not engaged in physical activities even to the extent I had used to before.”* (FGD_5_; P_2_, 52 years, Male)

*“Earlier I used to ride my bicycle for about 3 km. But now my eye sight is very poor and therefore cannot do cycling. Hence now I am only engaged in walking as a physical activity.”* (FGD_4_; P_3_, 65 years, Male)

None of the participants were aware of or discussed the importance of warming up, stretching, resistance exercise, and cool down activities. They also did not discuss possible adverse effects of physical activity and precautions to be taken before physical activity for a patient with diabetes.

## Discussion

To our knowledge the present study is the first qualitative study to evaluate the perception about diet and physical activity among Sri Lankan adults with diabetes mellitus. Studies have shown that Sri Lankan adults consume high levels of carbohydrate, fat (mainly saturated) and sodium, and low levels of protein and dietary fibers, which may have detrimental effects on health and be related to the current epidemic of diabetes. Furthermore, a nationwide survey on physical activity among Sri Lankan adults has demonstrated that 11 % are physically inactive, while physical inactivity was associated with central obesity, diabetes, hypertension and metabolic syndrome [16]. In the present study most patients with diabetes were aware of the importance of diet and physical activity in relation to their disease. However, they emphasized many difficulties faced when implementing these practices, especially social circumstances. Their food choices were mainly determined by the choices of those in the family, including non-diabetic healthy individuals. Lack of autonomy regarding food choices and the need to conform to family practices was a key issue identified by many participants. This has not been previously described in the literature, even in other South Asian countries. Previous studies on minority communities in western countries have highlighted different social issues, such as lack of accessibility to healthy food and ease of access to fast foods [18, 19]. Hence, it is important that health care professionals are sensitive to these ‘special’ social issues, when implementing behavioural change programmes aimed at modifying diet and physical activity. It is important to identify such factors early and offer alternatives and/or solutions, in order for the intervention to be successful.

Furthermore, there was lack of clarity and a high variability in opinion with regards to correct dietary practices, especially with regards to serving sizes and frequency of meals. In addition, most participants demonstrated a genuine interest in seeking high quality, reliable, easily understandable and readily accessible dietary advice. This emphasises the clear need for development of an effective and meaningful national dietary guidelines for patients with diabetes. It is important to have such locally developed and culturally sensitive guidelines, since some of the main food items are different from those found elsewhere in the world. A variety of national and international bodies have promoted the involvement of consumers as stakeholders in development of such dietary guidelines [20]. Such guidelines should also aim to tackle common dietary myths encountered in the local population, and qualitative studies are important to generate necessary evidence.

In the present study we also came across several such dietary myths, commonly prevalent amongst the population with diabetes. This includes the concept that certain food items are inherently “good” or “bad” for patients with diabetes, a message sometimes conveyed to these patients by media and in some instances by health care professionals. Other common misperceptions such as complete avoidance of sugar and fruits, also stems from the above concept. As mentioned earlier the total diet or overall pattern of food eaten is the most important focus of a healthful eating style. All foods can fit within this pattern, if consumed in moderation with appropriate portion size and combined with regular physical activity [11]. Nutrition messages are more effective when focused on positive ways to make healthful food choices over time, rather than individual foods to be avoided [12].

Most participants also came up with a list of ‘special’ food items that they felt has helped them to control blood sugar in addition to their medications. Some of the food items identified were: Fenugreek Seeds (‘Uluhal’), *Coscinium fenestratum* or yellow vine (‘Venivel gata’), curry leaves and powder and bitter-gourd. Patients have derived such information commonly from media, native medicine and other patients. Although there is some evidence in the scientific literature (mainly from *in-vitro* and animal study data) regarding the benefits of these food items, there is a lack of scientific consensus at present to support or refute these claims in humans [21, 22]. Furthermore, most of these claims are yet to be verified/refuted by properly conducted large scale randomized clinical trials. However, it is important that health care professionals take steps to ensure such practices do not interfere with the patients’ current treatment. Furthermore, it may also benefit science if future studies are developed to scientifically evaluate such strongly advocated claims.

Physical activity is proven to help prevent and control diabetes, both independently, and through weight control [23]. Most study participants were aware about the importance of being physically active. However, there was lack of consensus and clarity with regards to type, duration, timing and frequency of physical activity. Many cited walking when describing their physical activity types but the amount of walking varied amongst the participants. For most participants’ who engage in physical activity (including walking) it was an irregular commitment. Patients claimed to have only received vague and non-specific guidance about physical activity from health professionals and from other sources such as media. It is important that health care professionals understand the importance of physical activity, and have the ability convey the same to their patients. Development of local guidelines that are socially and culturally sensitive, with involvement of all stakeholders, including patients, might be the first step in bridging the knowledge gap. The main perceived barrier to regular physical activity was health factors related to complications of diabetes and their fear of such complications getting aggravated during physical activity. Awareness regarding these barriers and appropriate interventions are important in maintaining motivation and commitment in physical activity interventional programs.

This study helped to fill a nationally recognised research gap. The qualitative methods used were rigorous and the study suggested some new findings regarding perceptions on diet and physical activity amongst Sri Lankan adults with diabetes. There is also need for further research to understand the non-specific guidance and the perceived lack of encouragement from health professionals identified in this study. There are several limitations in the present study. The majority of respondents were Sinhalese (72 %) in ethnicity. However, there was a mixture of ethnicities and faith groups in each FGD and this could have affected the interactions within groups. Another weakness of the study may be the generalisability of the findings. The group of people interviewed were recruited from one clinical centre and may not be representative of the wider Sri Lankan population with diabetes. Previous nationally representative studies have shown that the mean age of the diabetes population of Sri Lanka is 55.0 ± 12.4 years (present study – 61.2 ± 9.9 years) and that 35.7 % were males (present study – 46 % males) [24, 25]. The majority of the population (83.6 %) are Sinhalese in ethnicity (present study – 72.0 % Sinhalese) [25]. The prevalence of diabetes related complications in the study population is not known. The presence of complications also could have influenced their dietary preferences and knowledge.

## Conclusions

Despite understanding the importance of dietary control and physical activity in the management of diabetes, adherence to practices were poor, mainly due to lack of clarity of information provided. There were many myths with regards to diet, some of which have originated from health care professionals. More evidence is needed to support or refute the claims about ‘special’ foods that the participants believe as being good for diabetes.

## Additional file

Additional file 1:
**Interview Guide for Focus Group Discussions.** (DOCX 20 kb)
