# Supplementary material for: Knowledge and perceptions about diet and physical activity among Sri Lankan adults with diabetes mellitus: a qualitative study
Source: BMC Public Health. 2015 Nov 23;15:1160. doi: 10.1186/s12889-015-2518-3 (PMC4657222; doi:10.1186/s12889-015-2518-3)
Supplement: Additional file 1: — Interview Guide for Focus Group Discussions. (DOCX 20 kb) [file 12889_2015_2518_MOESM1_ESM.docx]

**Interview Guide for Focus Group Discussions**

**Introduction**

Thank you for accepting our invitation and taking part in this interview. The purpose of this study is to acquire information about diabetes from viewpoint of the patients’. This can be used to enhance your knowledge on health and can improve the quality of life. I will ask you a set of questions that will cover various aspects of your personal information related to diabetes. You may respond to these queries in any way you feel comfortable. It is perfectly fine with us if you do not want to respond. We would like to tape record the interview. The recording will be kept confidential and will be shared only with the research team.

**Personal Information**

Reference No -

Age -

Gender -

City -

Marital status -

Occupation -

Education -

Duration of diabetes -

Other diseases -

**Theme - Patient’s perception on diabetes mellitus**

**Component - General idea**

Question - What do you know about diabetes?

Probes:

- What are the risk factors for getting diabetes?
- What did your doctor say as the reason for getting diabetes?
- Do your family members have diabetes?
- What did you feel after diagnosis of the disease?
- How is the support given by your friends and others?
- Do you think that you have risks for getting other diseases because of diabetes?

**Component - Diet**

What is the importance of diet on diabetes?

Probe:

- Have you changed your normal dietary habits because of diabetes?
- What are the changes you have done in your meal?
- Is there anything you taking other than your normal food because of diabetes?
- Have you restricted any food?
- If so what are they?

**Component - Physical activity**

What is the importance of exercise for diabetes?

Probe:

- Do you think there is a relationship between exercise and diabetes?
- What are the exercises you normally engage in?
- For how long do you engage in exercises?
- What is your purpose of doing that?
